# Advances in Ocular Drug Delivery Systems

**DOI:** 10.3390/pharmaceutics13091383

**Published:** 2021-09-01

**Authors:** Armando Silva-Cunha

**Affiliations:** Faculdade de Farmácia, Universidade Federal de Minas Gerais, Belo Horizonte 31270-901, Brazil; armando@ufmg.br

There have been major advances in the treatment of eye diseases over the last years. Novel therapeutic agents, ranging from small molecules to gene therapies, are available in the market to treat diseases that affect both the anterior and posterior segments of the eye, while several other drugs are at different phases of clinical trials. Simultaneously, new drug delivery systems that allow the transport of therapeutic concentrations of substances to specific targets of the eye are being studied. Despite these advances, the main challenge remains in the need for alternatives for the treatment of chronic eye diseases that could increase the long-term efficacy and avoid the inconvenience of monthly intravitreal injections or daily and repeated administrations of eye drops.

This Special Issue of Pharmaceutics highlights some recent advances focusing on the developments in ocular drug delivery, especially in relation to sustained delivery systems. Moreover, the topics published as “Articles” in this Special Issue describe the development of new nanosystems for both topical and intraocular ophthalmic application, these include: lipoplexes, polymersomes, pullulan conjugates, lipid nanoparticles and nanomicelles.

A review paper was also published in this Special Issue entitled “Formulation considerations for the management of dry eye disease”. This is an interesting literature review developed by Agarwal et al. [1] about different aspects of the formulation challenges for the management of dry eye disease (DED), a chronic multifactorial disease affecting thousands of people around the world. This review highlights the challenges typically faced in eyedrop development intended for the management of DED. The various artificial tear supplements currently on the market, their mechanisms of action, as well as their application, are discussed. Furthermore, formulation strategies generally used to enhance ocular drug delivery, their advantages and limitations, as well as their application in commercially available DED eyedrops are described.

Experimental results on the recent advances in lipoplex technologies were published in this Special Issue. Lipoplexes are formed by the spontaneous binding of nucleic acids to cationic liposomes via electrostatic interactions and they are a promising strategy as a drug delivery system (DDS) to the ocular route. However, recent studies demonstrated that the positive charge of the cationic lipoplex could be detrimental for its mobility and induce aggregation in the different ocular tissues. Strategies to overcome these limitations are described in two works in this Special Issue. Both use particle coating technology to change the lipoplexes’ properties and increase their cellular uptake. One, using corona protein (PC) as a coating material, was published by Astarita et al. [2], and the other, using hyaluronic acid, was reported by Ribeiro et al. [3]. The PC is a layer of biomolecules that forms around the DDS in physiological environments by noncovalent interaction. The PC changes the DDS’s physical–chemical properties, providing them with a completely novel biological identity. Astarita et al. explored the interaction between mucins (MUC) and four formulations of lipoplexes with different lipid compositions. They demonstrated that exposing lipoplexes to a MUC-enriched medium led to the formation of MUC-coated lipoplexes with different sizes and surface charges. These results are the proof of concept that coating DDS with artificial PC may be an innovative strategy for efficient corneal drug delivery. The authors showed that artificial coronas were able to promote massive cellular uptake of lipoplexes by primary corneal epithelial cells.

In the same way, Ribeiro et al. [3] used hyaluronic acid (HA), an anionic polymer, to coat cationic lipoplexes and then the authors investigated the safety and efficiency of the HA-coated cationic lipoplex (HA-LIP) to deliver caspase-3 siRNA in retinal neurodegeneration rat models by retinal light injury. In this work, it was demonstrated for the first time, in vivo gene silencing in the retina using HA-LIP to deliver siRNA to retinal cells. Overall, the results indicated that HA-LIP could successfully deliver siRNAs to reduce the production of proteins involved in the apoptotic cascade of retinal neurodegeneration. So, new possibilities for the treatment of many retinal diseases using nonviral vectors carrying siRNA, especially disorders that involve photoreceptor death, were claimed by the authors.

Intravitreal injection (IVT) delivers the entire drug dose to the vitreous and is one of the best alternatives for the pharmacological treatment of some retinal diseases, but this procedure requires skill, causes discomfort to the patient, and is required frequently. Therefore, the prolongation of the IVT intervals is desirable and, in order to overcome these obstacles, Junnuthula et al. [4] published a very interesting work. The authors synthesized and characterized block copolymers of poly (ethylene glycol), poly (caprolactone), and trimethylene carbonate. These polymers self-assembled to polymersomes and polymeric micelles. These nanocarriers were stable and diffusible in the vitreous. Pharmacokinetics of the intravitreal nanocarriers in rabbits were successfully evaluated using in vivo fluorophotometry. This is a noninvasive method used to quantitate the intravitreal pharmacokinetics of a soluble antibody and it has never been applied to study intravitreal kinetics of nanomedicines. In this work, the authors observed that the polymersomes retain in the vitreous for several months, reaching the retina and targeting the optic nerve head region. Polymersomes are a promising injectable formulation platform for long acting and targeted IVT drug delivery. According to the authors, the advantages of polymersomes, when compared to other nanocarriers, include the lack of harsh manufacturing conditions, encapsulation of both hydrophilic and lipophilic drugs and versatile options in terms of particle size, charge, rigidity, shape, and drug release.

Age-related macular degeneration (AMD) is one of the most common diseases that impacts the posterior segment of the eye, affecting thousands of people worldwide and causing severe vision impairment and blindness. AMD is characterized by an increase in reactive oxygen species (ROS) and proinflammatory cytokines in the retinal pigment epithelium cells (ARPE-19). The current pharmacological treatment of wet AMD includes the IVT of anti-VEGF monoclonal antibodies (mAb) that inhibit the interaction between VEGF-A and VEGF receptors blocking the angiogenesis process. However, as the vitreous half-life of the mAb is low, repeated IVTs are needed, leading to a high risk of complications and resulting in poor patient compliance.

Tacrolimus (TAC) is a potent immunosuppressive macrolide drug used for atopic dermatitis and rheumatoid arthritis. It is a hydrophobic drug with a Log *p* value of 2.7 and a molecular weight of 804 g/mol. The drug has also been shown to prevent early retinal neovascularization in mice ocular tissues which were treated with streptozotocin-induced diabetic retinopathy. In this context, Gote et al. [5] described in their article the development and optimization a formulation containing TAC to be used both by IVT or topical administration for wet AMD. In this study, the authors have utilized a mixture of two polymers: PEG-hydrogenated castor oil-40 (HCO-40) and octyxonyl-40 (OC-40), to obtain a nanomicellar formulation (NMF) and the optimized formulation showed a particle size below 20 nm and a neutral zeta potential for maximum uptake in ocular cell lines. The NMF, due to their unique structure, can encapsulate highly hydrophobic drugs and enhance their solubility, which can facilitate drug penetration and its effective delivery to the target tissue. In fact, according to the authors, the TAC-NMF developed demonstrated excellent trans-well permeability in the in vitro dual-chamber eye model, biocompatibility and, in an in vitro bioassay study using retinal cell lines pretreated with retinotoxin, the potential of the formulation to reduce the level of inflammatory markers and ROS. These results along with the immunosuppressive effect of tacrolimus could prove to be a beneficial strategy for AMD. This could advocate the application of TAC-NMF as a promising strategy for back of the eye disorders.

Supramolecular polysaccharide-drug conjugates are interesting systems for intravitreal drug delivery as they can be chemically manipulated by conjugation strategies to tailor their biopharmaceutical properties. Accordingly, polysaccharide conjugates can be designed to yield prolonged residence time, extended drug release and cell targeting. Pullulan is an interesting platform to produce this kind of conjugate for drug delivery and it is a polysaccharide produced by Aureobasidium pullulans that possesses the main requisites for invasive administration: biodegradability, water solubility and biocompatibility. In this context, Kicková et al. [6] developed a new pullulan-dexamethasone conjugate as a potential intravitreal drug delivery system. Dexamethasone was selected as a model drug since it is a hydrophobic corticosteroid used as an anti-inflammatory and immune suppressing drug in ophthalmology. A synthetic procedure for pullulan activation and dexamethasone conjugation was reported. In vitro studies with ARPE-19 showed no toxicity of the conjugates in the cells. Moreover, intravitreally injected pullulan conjugates may release dexamethasone both in the vitreous and within the ocular cells after internalization of the polymer conjugate. Thus, pullulan-dexamethasone showed prolonged drug release in the vitreous as a result of the slow cleavage of the hydrazone linker used to conjugate the drug to the polysaccharide backbone.

The last paper of this Special Issue, written by Chirio et al. [7], describes the development and stability studies of lipid nanoparticles (NPs) as a carrier for the bevacizumab (BVZ) designed for the treatment of ocular pathologies related with neovascularization process. BVZ is another anti-VEGF mAb specially indicated for the treatment of diabetic and degenerative retinopathy, currently used “off-label” by IVT for many months or even years, because its suspension or discontinuation may cause the recurrence of the neovascularization process. According to the authors, the procedure developed to obtain the spherical BVZ-loaded NPs intended for IVT, named “cold dilution of microemulsions”, is a simple and highly efficient method, employing highly biocompatible substances permitted for parenteral use. The study also focused on the biochemical and biophysical stabilities of BVZ after entrapment in NPs and several physicochemical techniques were used for this purpose. The biocompatibility was assessed by in vitro cell compatibility studies using the ARPE-19 cell line. Together, the results obtained have shown that a stable BVZ-loaded system was obtained, the drug was released slowly from the lipid matrix and that this system is biocompatible. Thus, the BVZ-loaded NPs system could be useful to reduce the IVT administration frequency, minimizing adverse effects and ameliorating the patient’s therapy acceptance.

As a conclusion of this Special Issue, “Advances in Ocular Drug Delivery Systems”, I hope the papers published here have shown some recent advances in ocular drug delivery system developments. This is a fascinating area of research which is constantly evolving and has a worldwide significance.
